# Phylogenetic Diversity Theory Sheds Light on the Structure of Microbial Communities

**DOI:** 10.1371/journal.pcbi.1002832

**Published:** 2012-12-20

**Authors:** James P. O'Dwyer, Steven W. Kembel, Jessica L. Green

**Affiliations:** 1 Santa Fe Institute, Santa Fe, New Mexico, United States of America; 2 Institute for Ecology and Evolution, University of Oregon, Eugene, Oregon, United States of America; 3 Départment des sciences biologiques, Université du Québec à Montréal, Montreal, Quebec, Canada; University of Toronto, Canada

## Abstract

Microbial communities are typically large, diverse, and complex, and identifying and understanding the processes driving their structure has implications ranging from ecosystem stability to human health and well-being. Phylogenetic data gives us a new insight into these processes, providing a more informative perspective on functional and trait diversity than taxonomic richness alone. But the sheer scale of high resolution phylogenetic data also presents a new challenge to ecological theory. We bring a sampling theory perspective to microbial communities, considering a local community of co-occuring organisms as a sample from a larger regional pool, and apply our framework to make analytical predictions for local phylogenetic diversity arising from a given metacommunity and community assembly process. We characterize community assembly in terms of quantitative descriptions of clustered, random and overdispersed sampling, which have been associated with hypotheses of environmental filtering and competition. Using our approach, we analyze large microbial communities from the human microbiome, uncovering significant variation in diversity across habitats relative to the null hypothesis of random sampling.

## Author Summary

Microbial diversity analyses have revolutionized our knowledge of the microscopic world, from terrestrial and marine to human and urban environments. This growing field rests on the evolutionary relatedness of organisms, and at its frontier is the inference of ecological processes from phylogenetic diversity. However, the rapidly reducing cost of sequencing means that computational analysis of phylogenetic data is becoming increasingly intractable. We develop a new analytical method to address this issue, providing a computationally-efficient way to compare local phylogenetic diversity to a sample from a regional pool of organisms, under a given ecological process. Our approach has both pragmatic and far-reaching applications. Until now investigators have lacked even an analytical method to compare the diversity of unequally-sized communities without throwing data away, while on a deeper level our theory provides a new framework for connecting phylogenetic data to a wide range of ecological processes. As an application of our approach, we use our methods to distinguish between random, clustered and overdispersed sampling for human microbiome habitats. Finally, we identify a new, phylogenetic analogue of the widely used taxonomic measure of diversity, the Species Abundance Distribution, and we find that it has consistent behavior across microbiome habitats.

## Introduction

Microbial ecology has been advancing at a rapid pace, but understanding the processes driving microbial community structure remains a challenge [1]. The first step towards identifying these processes has been to document microbial biodiversity, and the use of phylogenetic methods has been stimulated by the abundance of genomic data harvested from microbial communities [2]. Phylogenetic measures of diversity have been proposed as a more accurate representation than taxonomic diversity of community trait and functional diversity, and are therefore a potentially more relevant starting point for quantifying and understanding microbial communities [3]. Phylogenetic approaches have been used to quantify microbial diversity along environmental [4] and elevational gradients [5], [6], across distinct habitat types [7]–[10] and for different experimental treatments, for habitats ranging from marine and freshwater, to soil, indoor and outdoor air, to the human body [11].

Despite this success in documenting patterns of phylogenetic diversity in a broad range of contexts, our ability to translate these patterns into processes has been hampered by a lack of phylogenetic theory. The difficulty in formulating quantitative hypotheses impacts even the simplest of questions: which of two microbial communities is more phylogenetically diverse? If one community is larger than the other, we cannot answer this basic question without a theoretical hypothesis for the way we *expect* phylogenetic diversity to increase with community size. More generally, the lack of quantitative, analytical theory has made it difficult to address the relative importance of ecological processes of environmental selection, competition, dispersal and stochasticity in a given community. We have qualitative hypotheses and computational approaches to assess the impact of these processes, but there is no overarching, analytical framework within which to compare them.

Phylogenetic theory can address both of these issues: the pragmatic problem of comparing phylogenetic diversity in different communities, and the larger question of inferring ecological processes from phylogenetic patterns. In this manuscript we develop a way to cast many different assembly processes in a common framework, centering around the comparison of a local community of co-occuring organisms with a sample from a regional, metacommunity of organisms. We draw from the sampling theory of taxonomic diversity [12]–[14] and our analysis rests on a new way of characterizing a metacommunity phylogenetic tree, which we term the Edge-length Abundance Distribution. This distribution is a new phylogenetic analogue of the classic taxonomic Species Abundance Distribution.

As a proof-of principle application of our framework, we focus on publicly-available human microbiome data [11] and explore three questions centering around the comparison of microbiome phylogenetic diversity with a null hypothesis of random sampling from a metacommunity. First we document patterns across different microbiome habitats and different subjects, and identify a power-law pattern for the Edge-length Abundance Distribution. Species Abundance Distributions have been proposed to have a log-normal distribution in many ecological communities [15], [16], including microbial systems [17], and our work puts this observation on a new, phylogenetic footing. Second, we examine the effect of metacommunity scale on distinguishing different community assembly hypotheses [18], [19]. We compare the diversity of body habitats to a null hypothesis of random sampling from two different definitions of the metacommunity, one significantly larger than the other. We see a clear impact of metacommunity size on the phylogenetic diversity of body habitats relative to the null hypothesis. Finally, we address the question of whether the microbiome of a given human subject is consistent with the null hypothesis of random sampling, and find that while whole microbiome diversity for a given subject is typically much lower than a random sample from the metacommunity, this hides a wide range of different behaviors for the distinct habitats within that subject. We think of this as exploring the impact of local community resolution within the human body: different levels of resolution reveal more complexity.

## Results

### Phylogenetic Diversity Reflects Community Assembly

Phylogenetic Diversity (PD) has been defined as the total branch length connecting all organisms in a phylogenetic tree, and provides a natural phylogenetic analogue of taxonomic diversity [20]. Similarly, the UniFrac distance measure quantifies the overlap in phylogenetic branch length of two communities, and tells us how similar or different those two communities are [21]. PD and UniFrac provide convenient measures of microbial diversity that do not rely on the ability to identify or enumerate microbial species, and are also amenable to exploring phylogenetic versions of classic biogeographical patterns of alpha and beta diversity.

Comparing observed patterns of phylogenetic diversity to the patterns expected under various null models provides both a normalization to take into account the difference in sample sizes across different habitats or treatments [22], [23], and also a connection between patterns of phylogenetic diversity and the processes underlying them. Given information on the evolution of ecologically important traits [24], [25] and the phylogenetic relatedness of organisms in local communities, an ‘ecophylogenetic’ framework can potentially provide insights into the relative importance of processes such as dispersal, competition, filtering, or drift [26]–[30]. So far, these approaches to evaluating phylogenetic diversity against null models have relied on ‘brute force’ sampling, where a randomizing algorithm is used to sample tips from a phylogenetic tree, and the process is repeated many times to infer expected phylogenetic diversity for a sample of a given size. This approach becomes intractable for large phylogenetic trees, and must also be repeated for each additional hypothesis and sample size.

**Figure 1:**
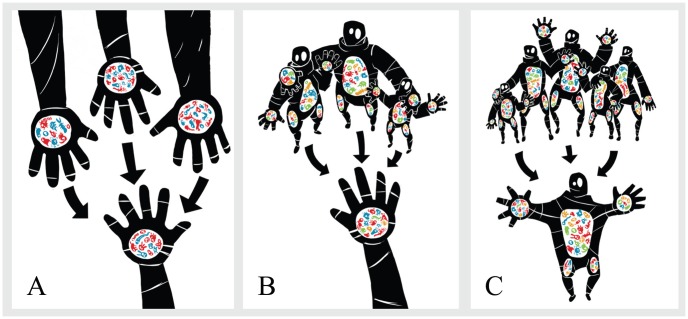
The local community and metacommunity framework casts local biodiversity of coexisting species in terms of a sampling process from a larger reference pool, or metacommunity. In (A), (B) and (C) we adapt this framework for a microbial community, the human microbiome, for different definitions of the local community and the reference metacommunity. (A) shows microbiota from a single body habitat, thought of as a sample from the pool of microbiota found in the same habitat on different human subjects. (B) shows the same local community, but thought of as a sample from all microbiota across multiple humans. Finally, the local community in (C) is all microbiota from a single human subject, while the metacommunity is again microbiota from multiple habitats across multiple humans.

We have developed a new, analytical approach to address these problems. Our conceptual framework links a local sample of individual organisms, a regional pool or metacommunity, and processes connecting these two scales. This perspective has a long history in ecological theory [31]–[33], and has been recently advocated as an appropriate framework for the ecology of symbiont systems [34]. Figure 1 outlines the conceptual overview for this local and metacommunity framework, with explicit examples in the context of the human microbiome. Within our framework, the community assembly processes linking these local and regional scales could be either mechanistic, directly drawing on a process such as dispersal; or phenomenological, characterizing general patterns in statistical terms. The phylogenetic diversity literature has most often focused on the latter, comparing observed PD with the hypotheses of random, clustered and overdispersed sampling. We therefore focus on these sampling schemes in this manuscript, but our framework is potentially generalizable to specific community assembly mechanisms such as dispersal limitation [14]. The methods described in this paper have been implemented in software and will be available in version 1.5 of the picante R package [35].

#### Expected phylogenetic diversity and variance

**Figure 2:**
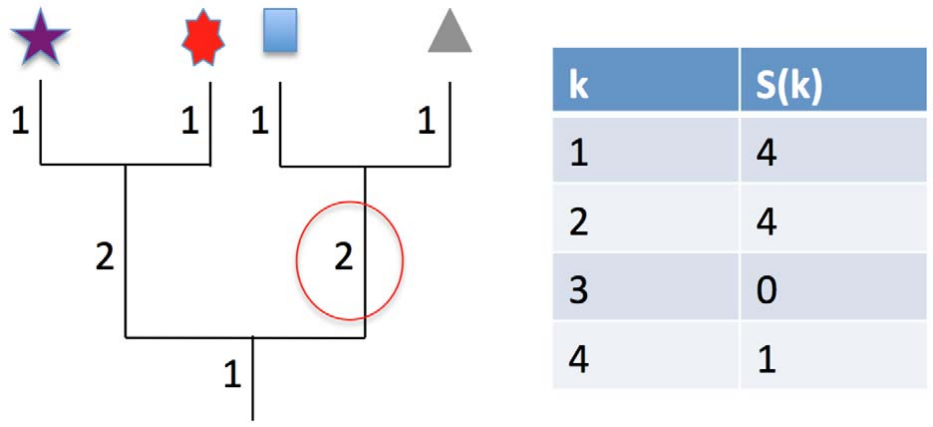
The Edge-length Abundance Distribution, 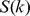, for a community with four individuals. All edges are labeled by their length, and the table shows the total edge-length summed across all edges with a given number of upstream tips. Number of tips appears in the left column, and the total edge-length associated with that number of tips, 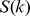, is shown in the right column. For example, the circled edge has length two, and has two tips downstream, and therefore contributes length 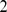 to 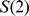.

Our central result is an analytical method to obtain the expected phylogenetic diversity of a local sample from a larger community. In the Supplementary Information we derive the following expression for expected phylogenetic diversity, 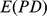:
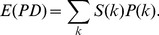
(1)Each term in the sum includes all clades in the metacommunity tree which have 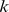 tips. 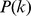 is the probability that a clade with 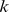 tips will have *at least one* representative in the sampled tree, and depends on the choice of sampling process and the local community size. The function 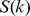 only depends on the structure of the metacommunity tree, and is defined as the sum of the branch length of all edges with 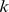 descendent tips. This is a new way to characterize the phylogenetic structure of the metacommunity tree, which we term the Edge-length Abundance Distribution (EAD). We give a description in Figure 2. We also provide an analytical method to compute the variance in sampled PD, discussed in further detail in the Supplementary Information.

**Figure 3:**
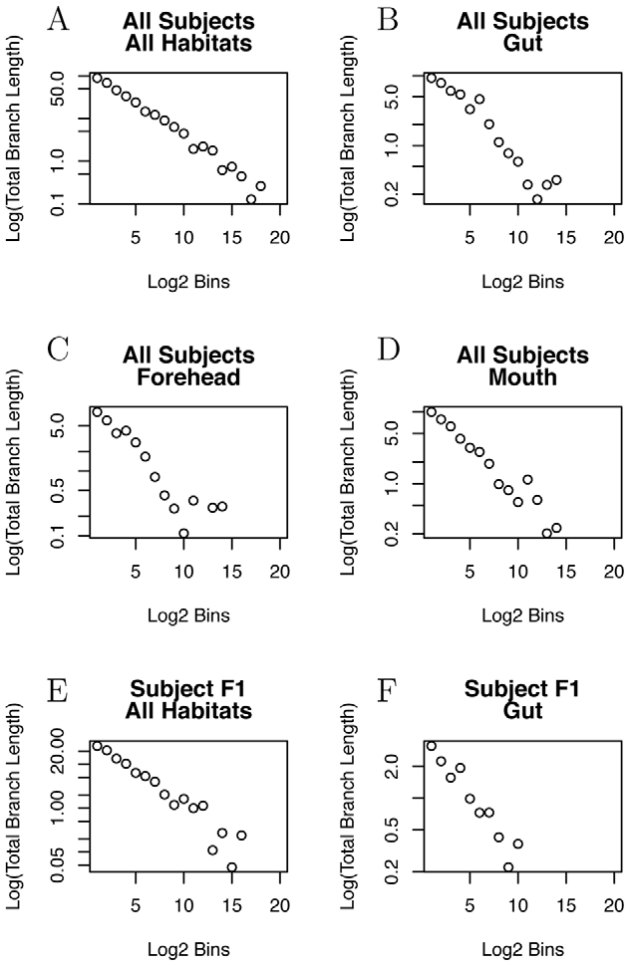
The Edge-length Abundance Distribution (EAD) follows an approximately power-law distribution. Here we compute the EAD for trees inferred from 16S bacterial microbiome sequences using FastTree [47], for sequences obtained from 7 human subjects across 26 different habitats on a single sample date [11]. On the y-axis is total edge-length summed across all edges with a given number of downstream tips (see Figure 2) and on the x axis is number of tips grouped into 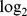 bins, a method commonly used in plotting Species Abundance Distributions [15]. (A) Sequences from all individuals across all body habitats (B–D) Sequences taken from different habitats pooled from all seven subjects. (E–F) Sequences taken from a single subject.

The EAD plays an identical role in our sampling framework to the Species Abundance Distribution (SAD) in taxonomic sampling theory [12], [13]. But what does this distribution look like? For the human microbiome we give several examples of the EAD across different subjects and habitats in Figure 3 and find that the distribution displays approximately power law behavior. The Species Abundance Distribution has been proposed to take a near-universal log-normal form in microbial communities [17], and our preliminary results suggest that a power-law distribution may be its phylogenetic analogue.

Random sampling has frequently been used as a null model in phylogenetic community ecology, but with the disadvantage of needing to take random samples computationally. For a binomial sampling scheme each individual in the metacommunity has the same probability 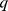 that of appearing in the sampled tree, so that no individual from the regional pool is more or less likely than any other to appear in a local community. This hypothesis leads to the probability
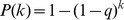
(2)that an edge with 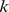 descendent tips appears in the sampled tree, so that
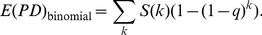
(3)Expected phylogenetic diversity arising from other commonly used sampling schemes can be cast in the same form: given the EAD for a metacommunity we have the expected PD as a function of sample size for a potentially vast range of community assembly processes.

**Figure 4:**
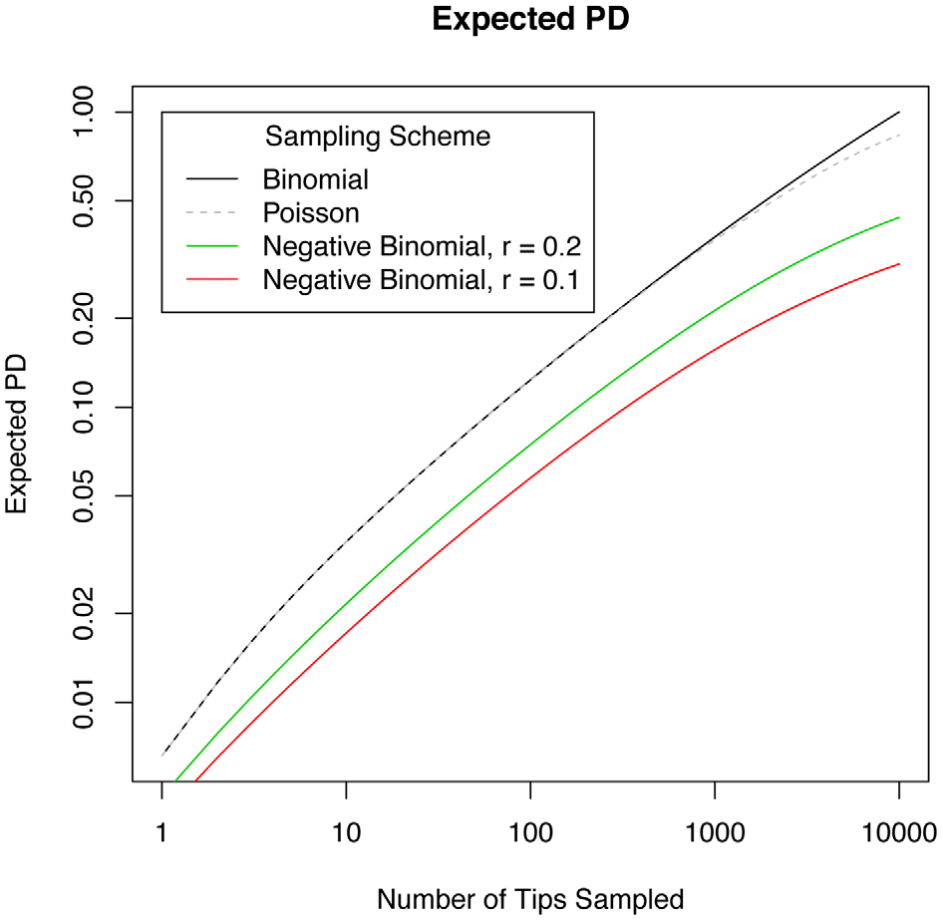
Expected PD for a sample taken from a theoretical metacommunity with a power-law Edge-length Abundance Distribution, 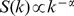 for various sampling schemes. As an approximation to the real communities in Figure 3, we take 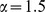. The resulting expected PD for Binomial sampling is close to a power law as a function of number of tips sampled, with 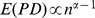. We show the corresponding result for Poisson sampling, representing sampling with replacement which is identical to the binomial result until sample size becomes large. Finally, we show two negative binomial schemes with different clustering parameters, 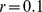, 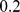 (see Supplementary Information). These parameters are consistent with different degrees of phylogenetic clustering, which leads in general to smaller sampled PD for a given number of tips.

We demonstrate our approach in Figure 4, where we make the choice of a power law function for the Edge-length Abundance Distribution, similar to those seen for the human microbiome communities in Figure 3. Binomial and Poisson sampling both correspond to the local community being formed by random sampling. The binomial case corresponds to sampling without replacement, while Poisson sampling corresponds to sampling with replacement. Negative binomial sampling can be parametrized to characterize either clustered sampling, where nearby tips are more likely to be sampled together, or overdispersed sampling, where sampling across tips is spread more evenly. The appropriate 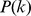 for binomial sampling is given above, while Poisson sampling and negative binomial are as follows:
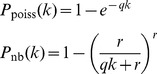
(4)The parameter 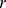 represents the departure from random sampling, with positive 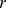 indicating clustered sampling, negative 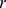 overdispersed sampling, while in the limit of 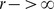 the negative binomial and poisson sampling are equivalent.

For binomial samples from a power-law EAD, we observe a power-law increase in expected PD with sample size, consistent with the power-law increase in expected PD with sample size observed in other kinds of community [36], [37]. Poisson sampling produces an identical pattern up to very large sample sizes where replacement becomes important. Finally, clustered sampling produces a similar pattern with lower overall PD for a given sample size.

#### Expected phylogenetic 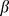-diversity: unequal sample size increases UniFrac

We can express the expected shared branch length for two local samples as:
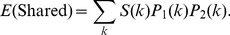
(5)Again, the EAD 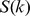 appears, but now we have multiple probabilities 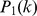 and 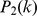 corresponding to the probability that a clade with 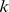 tips has at least one individual in local community 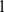 or 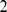. Under binomial sampling with probability 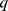 of each tip being sampled we have:
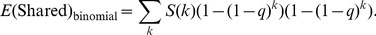
(6)It is more common to compute the unique fraction of branch length for two samples, known as the UniFrac distance, which depends on both this shared branch length, but also on total combined branch length across both samples. Identical samples have no unique branch length, and a Unifrac score of zero, while samples with no branch length in common have a UniFrac score of one.

In one sense UniFrac distance is *already* normalized: the UniFrac distance of two equally-sized, high diversity communities can be consistently compared with the score for two equally-sized low-diversity samples. What cannot be compared is the UniFrac distance of pairs of *differently*-sized samples. While the potential impact of unequal sample sizes on computing UniFrac distances has previously been recognized, a common solution has been to subsample [7] all samples down to the smallest size, with the drawback that some data is thrown out.

**Figure 5:**
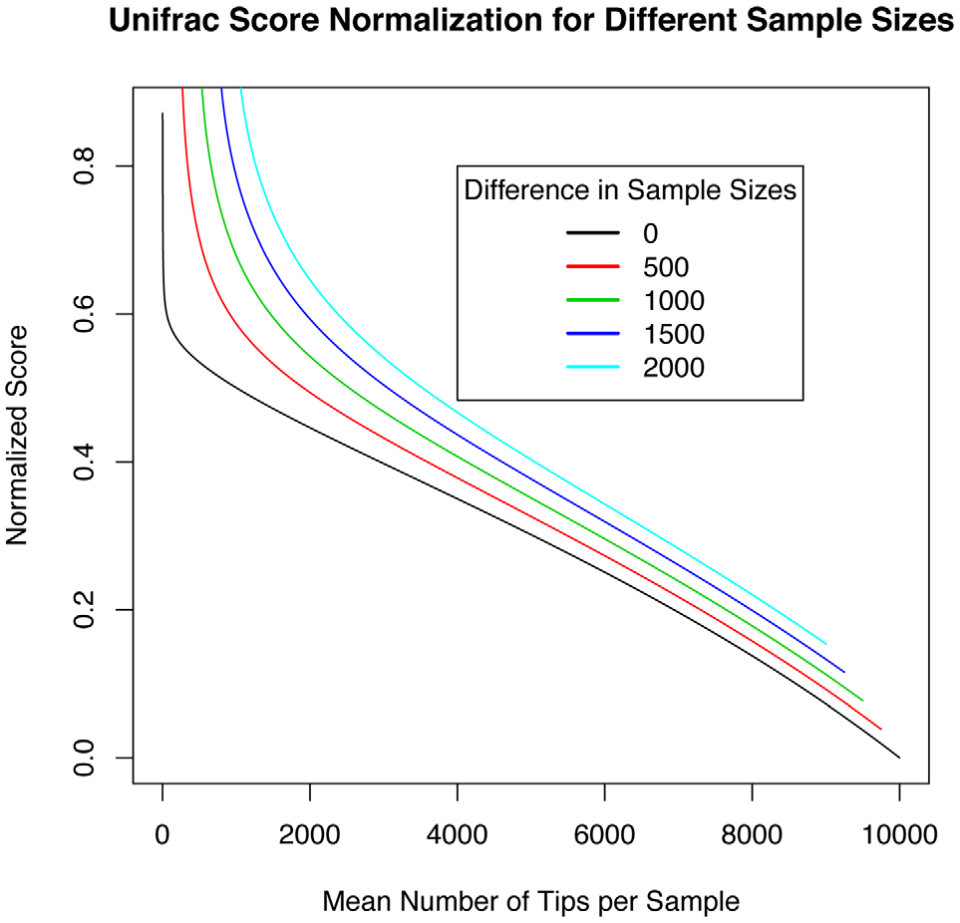
Unifrac score normalized for different sample sizes, for two random samples taken from the same theoretical metacommunity. The score is calculated in terms of expected shared branch length and expected total branch length for the two samples. The black line is for samples of the same size, and colored lines are for various differences in sample size, 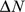. As 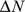 becomes a larger fraction of mean sample size, there is an increasing deviation from the expectation value for equal samples sizes, indicating that an appropriate normalization accounting for differing sample sizes is potentially crucial in computing phylogenetic beta diversity for differently-sized samples.

We can avoid this if we have a normalization for how UniFrac changes with unequal sample sizes. We use our sampling framework to normalize shared and total branch length to provide this normalization. In Figure 5 we plot this result, based on random, binomial sampling from a simulated metacommunity with power law EAD. We find that unequally-sized samples typically have a greater UniFrac normalization than two equally-sized samples from the same regional pool.

### Human Microbiome, Scale and Resolution

The human body is host to multiple microbial communities, whose combined total outnumbers our own cells by at least a factor of 10 [11], and a community ecology perspective may be essential for a full understanding of the impact of the human microbiome on human health [38]. We now apply our framework to such microbial communities, collected from 7 human subjects across 26 distinct habitats. This data provides an ideal testing ground to demonstrate the utility of our theoretical framework: the largest metacommunity tree we sample from has 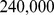 tips, each tip representing an individual sequence sampled in the study, which would render computational null model approaches intractable. Also, the different community types, subjects and scales allow us to address how our community assembly hypotheses depend on the choice of local community and metacommunity.

#### Comparing local community phylogenetic diversity with a null model of community assembly

We now focus on the deviation of microbiome communities from randomly assembled communities. The reason that random sampling is such a crucial baseline is that it lies between two distinct phylogenetic community assembly processes: local communities with lower PD than random are phylogenetically clustered, a signature of environmental filtering, while local communities with higher than random PD are phylogenetically overdispersed, indicating competitive interactions. The true relationship between clustering and overdispersion and these ecological mechanisms is likely more complicated than this simple description [39], [40], but it is a useful starting point [27].

To evaluate the structure of a local community with respect to a given community assembly hypothesis, we need to make an assumption about the pool of individuals or taxa that this local community can draw on: the metacommunity. The ‘true’ regional pool of organisms that a given habitat from a given subject draws upon is difficult to define unambiguously, and so our approach is to allow for various definitions of the metacommunity, and examine the sensitivity of our results on these definitions. This relevance of metacommunity definition has been highlighted before [18], where it has been shown that different metacommunity sizes can lead to different conclusions about the most important ecological mechanisms structuring local communities, and our approach allows us to explore this issue for large microbial metacommunities. Secondly, we also investigate the effect of local community resolution on community assembly hypotheses: how does dividing a local community into smaller, sub-communities impact our understanding of community assembly. In particular we focus on grouping together distinct habitats to form the whole microbiome for a given subject, versus looking at habitats individually, and the effect of these two choices on our conclusions about community assembly.

**Figure 6:**
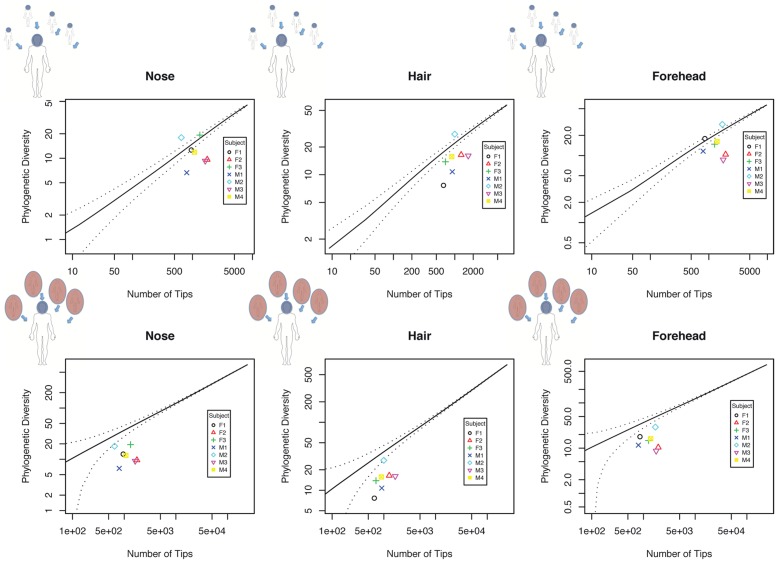
Changing metacommunity scale can change whether samples appear clustered or overdispersed. We show a comparison of actual PD with expected PD under random sampling for three habitats: nose, hair and forehead. Solid lines represent expected PD from random sampling as a function of expected number of tips, dashed lines represent an upper bound on the 95% confidence interval computed using the variance in sampled PD (see Supplementary Information). Points represent actual PD from each of seven human subjects on one sampling day, with subjects labeled by a letter (M or F indicating male or female) and an identifying number. On the top row the reference metacommunity is all reads from *that* specified habitat, pooled across all seven subjects, and on the bottom row the metacommunity is all sampled reads across all subjects *and* all habitats. Our central conclusion is that while most samples are consistent with phylogenetic clustering, irrespective of the metacommunity, a minority of communities are consistent with overdispersed sampling from the smaller metacommunity, but clustered sampling from the larger.

First, we find that PD increases as a power-law function of sample size. Our definition of a local community is all reads sampled from a single habitat, from a single subject on a single sample day. We plot the results for three habitats in Figure 6, for two different definitions of the metacommunity: (1) all sampled reads from that habitat, across all subjects, and (2) all sampled reads from all habitats, across all subjects. We note the behavior of expected PD, the solid lines in Figure 6: expected phylogenetic diversity as a function of sample size is close to a power law: this is precisely what we would expect for random sampling from a power-law EAD, just as we saw in Figure 3.

Next, we see that larger metacommunities make local samples more likely to appear clustered. There are clear differences between these two example habitats in terms of the range of PD across subjects. For metacommunity (1) we see that most samples are phylogenetically clustered, but several of them are overdispersed. In contrast, from metacommunity (2) *all* samples are consistent with phylogenetically-clustered sampling. This demonstrates the importance of metacommunity size on distinguishing between hypotheses of environmental filtering and competitive exclusion.

**Figure 7:**
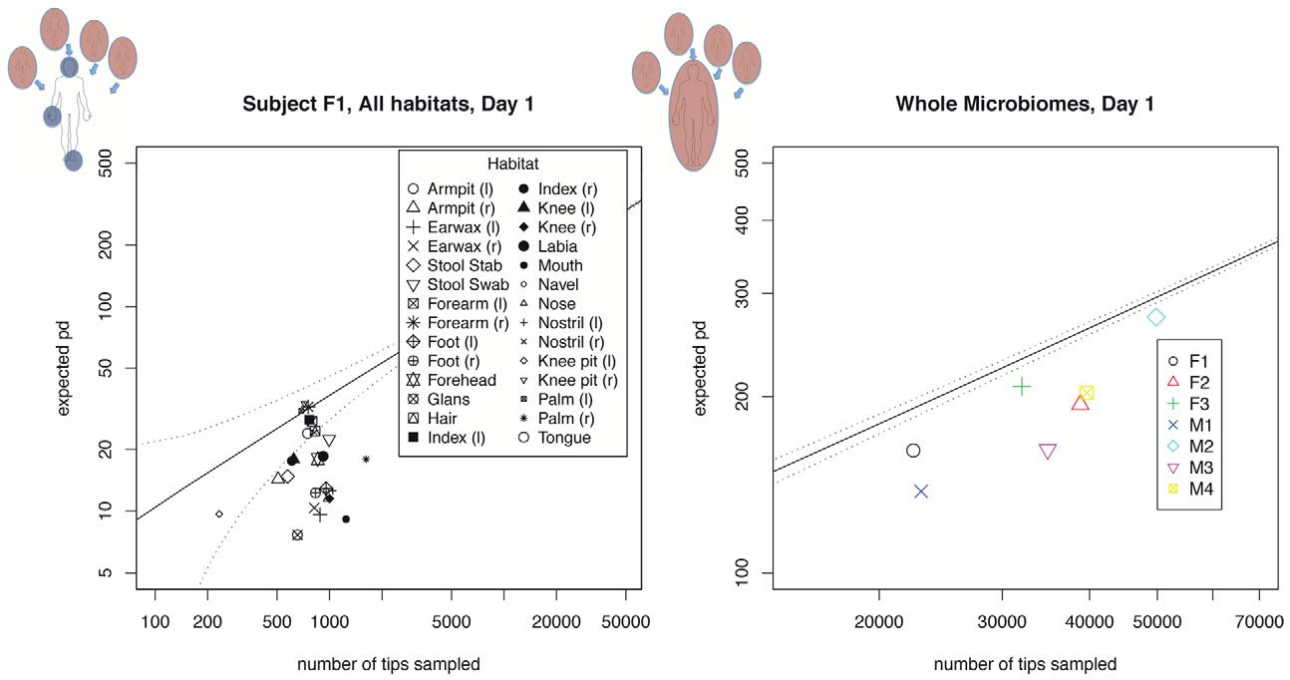
Increasing local community resolution reveals a diversity of clustered and overdispersed local samples. We show a comparison of actual PD with expected PD for multiple habitats and subjects. The lefthand panel shows the PD of all habitats sampled from subject F1, with the solid line indicating the expected phylogenetic diversity of a random sample from a metacommunity comprising all reads taken from all subjects, and the dotted line representing an upper bound on the corresponding 95% confidence interval. The right hand panel shows the total branch length for all reads sampled from each subject (again including F1 in black), with solid and dotted lines as on the left hand-side. When local samples are grouped into a single community for each microbiome, all microbiome communities are consistent with clustered sampling. On the other hand, we observe a wide range of variation in habitat PD relative to random sampling.

Finally, increasing Local Community Resolution reveals variation in local clustering and overdispersion. In Figure 7 we address the question of whether the microbiome of a given human subject is consistent with clustered, random, or overdispersed sampling. We consider local communities comprising all reads from a given individual, with metacommunities defined using all reads from all subjects on a single sample date. In Figure 7(B) we plot each subject as a single point, seeing that all individuals are consistent with clustered sampling. In Figure 7(A) we zoom in to one subject, plotting their microbiome PD as a cloud of individual habitats. Plotted as single points there is a significant spread in terms of PD with respect to random sampling, with some local communities overdispersed and some highly clustered.

## Discussion

In this manuscript we have developed a new, analytical method to quantify and distinguish different hypothesis for the phylogenetic structure of ecological communities. Our approach centers around a new characterization of phylogenetic tree shape, which we term the Edge-length Abundance Distribution (EAD), and we find that this distribution is analogous and complementary to the Species Abundance Distribution (SAD) in taxonomic sampling theory. We observe that the EAD follows a roughly power-law distribution across a number of communities within the human microbiome. Power-law patterns in the distribution of branch lengths have been observed before in phylogenetic trees [41], but while intriguing, the relevance of these patterns was not clear. What makes this pattern different is that, just like the SAD and taxonomic diversity, the definition of the EAD is not arbitrary: it has an essential role in connecting local and regional diversity. An important next step will be to identify what kinds of ecological mechanisms and constraints can give rise to particular types of Edge-length Abundance Distribution, including approximately power law distributions.

We applied this theoretical framework to investigate whether phylogenetic diversity (PD) of local communities from the human microbiome is greater or less than the expected PD for randomly drawn samples from a metacommunity. Local community PD lower than random has been associated with the hypothesis of environmental filtering, while local community PD greater than random (phylogenetic overdispersion) has been associated with competition and competitive exclusion [27]. Taking as our local communities the set of organisms sampled from a single habitat from a single human subject, we observed a wide range of variation across habitats and across subjects in terms of deviation from the random hypothesis. On the other hand, it is not clear yet how to quantitatively connect these deviations to ecological processes, indicating that sampling schemes with a direct connection to ecological mechanism may in the longer term be more relevant than the phenomenological sampling schemes we have explored in this manuscript.

Our results set the scene for a much more rigorous investigation of these issues, and we see three main future directions. For the first time, our framework has made the phylogenetic analysis of large microbial metacommunities analytically tractable, and we find that metacommunity size is highly relevant in our proof-of-principle analysis of human microbiome communities. This confirms the expectation that our conclusions about community assembly depend crucially on the definition of the metacommunity, and indicates the need for a very careful definition of the metacommunity to fully understand the processes structuring local phylogenetic diversity. Second, we have adapted tools from taxonomic sampling and applied them in a phylogenetic context, but this provides just the first steps towards developing a comprehensive theoretical toolbox for distinguishing hypotheses and predicting patterns. Directly characterizing ecological mechanisms, for example dispersal limitation, in terms of our phylogenetic sampling theory will provide a clearer connection between ecological process and phylogenetic patterns.

Finally, we have focused here on the total phylogenetic diversity of local communities [20]. This is the analogue of looking at total species richness alone as a way to distinguish between different hypotheses. In studies of taxonomic diversity, species abundances have provided a way to distinguish quantitatively between different types of community [15], [16], [42], [43], to extrapolate taxonomic diversity to scales far beyond our samples [17] and to connect taxonomic pattern and mechanistic processes more clearly [16], [33], [44]–[46] than using species richness alone. We do not yet have the overarching phylogenetic theory with which we can distinguish between environmental selection, competition, dispersal and stochasticity. But the application of our framework with mechanistically-based sampling schemes has the potential to put phylogenetic diversity on this same quantitative footing as taxonomic diversity, potentially allowing us to extrapolate PD from local samples to much larger scales, and to distinguish between different ecological hypotheses more effectively.

## Methods

Our methods are integrated into the body of the manuscript, primarily in the **Results** section. Additional methods and derivations are included in our Supporting Information **Text S1**.

## Supporting Information

Figure S1 **Hierarchical clustering for gut samples.** Figure S1 displays hierarchical clustering for gut samples. Distances are defined using the Unifrac metric, but normalized by expectation values corresponding to appropriately-sized random samples from the gut metacommunity.
(PDF)

Text S1 **Additional derivations.** Text S1 contains additional details of the methods used to obtain results in the main body of the manuscript. It provides a formal derivation for expected local community phylogenetic diversity from a given metacommunity Edge-length Abundance Distribution. It also provides similar derivations for the variance in expected PD and the expected shared and total branch length for two independent samples from a metacommunity.
(PDF)
