# Supplementary material for: Phylogenetic Diversity Theory Sheds Light on the Structure of Microbial Communities
Source: PLoS Comput Biol. 2012 Dec 20;8(12):e1002832. doi: 10.1371/journal.pcbi.1002832 (PMC3527210; doi:10.1371/journal.pcbi.1002832)
Supplement: Figure S1 — Hierarchical clustering for gut samples. Figure S1 displays hierarchical clustering for gut samples. Distances are defined using the Unifrac metric, but normalized by expectation values corresponding to appropriately-sized random samples from the gut metacommunity. (PDF) [file pcbi.1002832.s001.pdf]

Phylogenetic Diversity Theory Sheds Light on the Structure of Microbial Communities - Supporting Information

James P. O'Dwyer, Steven W. Kembel, Jessica L. Green

Figure S1

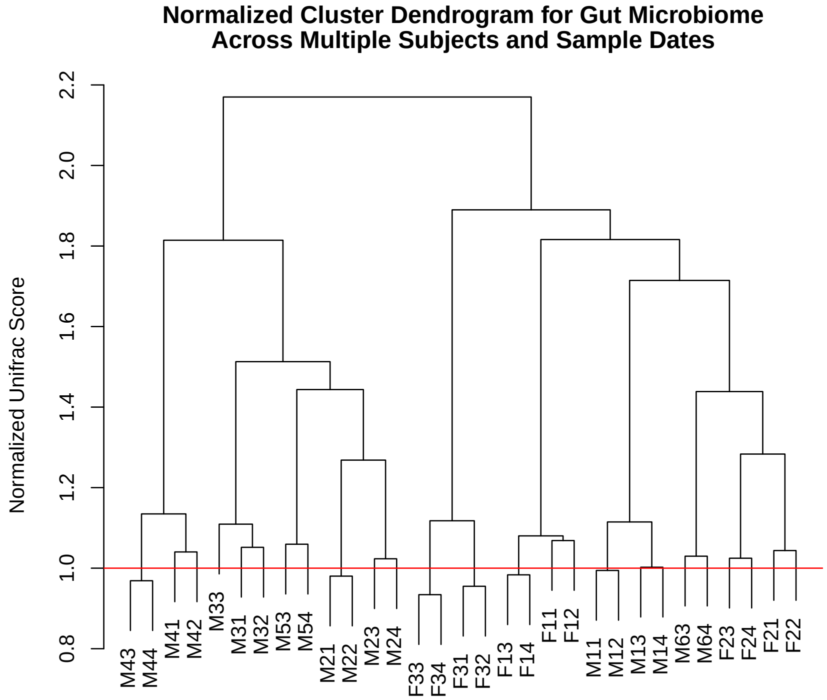

Figure S1 Hierarchical clustering for gut samples. Distances are defined using the Unifrac metric, but normalized by expectation values corresponding to appropriately-sized random samples from the gut metacommunity. Each sample is labeled by a letter (M or F indicating gender of the subject), and two numbers: the first number indicates subject, the second sample date. Sample dates 1 and 2 are on consecutive days, as are 3 and 4. The red line corresponds to a distance between a pair of samples which is equal to the unifrac score for two random samples, and so we see that a number of pairs of samples taken from the same subject on consecutive days are *more* similar than random samples. No other pairs are more similar than random.
