# Supplementary material for: Phylogenetic Diversity Theory Sheds Light on the Structure of Microbial Communities
Source: PLoS Comput Biol. 2012 Dec 20;8(12):e1002832. doi: 10.1371/journal.pcbi.1002832 (PMC3527210; doi:10.1371/journal.pcbi.1002832)
Supplement: Text S1 — Additional derivations. Text S1 contains additional details of the methods used to obtain results in the main body of the manuscript. It provides a formal derivation for expected local community phylogenetic diversity from a given metacommunity Edge-length Abundance Distribution. It also provides similar derivations for the variance in expected PD and the expected shared and total branch length for two independent samples from a metacommunity. (PDF) [file pcbi.1002832.s002.pdf]

Phylogenetic Diversity Theory Sheds Light on the Structure of Microbial Communities - Supporting Information

James P. O'Dwyer [1,2,*], Steven W. Kembel[2], Jessica L. Green[1,2]
1 Santa Fe Institute, Santa Fe NM USA
2 Institute for Ecology and Evolution, University of Oregon, Eugene OR USA
∗ E-mail: jodwyer@santafe.edu

Text S1: Additional Derivations

Expected Phylogenetic Diversity and the Edge-Length Abundance Distribution

We define sampled phylogenetic diversity to be the total branch length in a sampled tree, and we describe a sampled tree by a set of variables, g_i, where i runs over each edge in the metacommunity tree and each g_i can be either zero or one, corresponding to whether the edge also appears in the sampled tree.

We define the probability of finding a particular sampled tree as $P(g_1, g_2....g_{k_{\max}})$, where $k_{\max}$ is the number of edges in the metacommunity tree. An edge contributes S_i to the expectation value of total branch length if $g_i = 1$ and zero if $g_i = 0$, and so to obtain the expected phylogenetic diversity for a given sampling scheme we wish to compute the expectation value of the following variable:

$$H(g_1, g_2....) = \sum_i h_i(g_i) \tag{1}$$

where

$$\begin{aligned} h_i(1) &= S_i \\ h_i(0) &= 0. \end{aligned} \tag{2}$$

This expectation value is then (as for any arbitrary function of the variables g_i):

$$\begin{aligned} E(PD) &= \sum_{g_1, g_2 \cdots g_{k_{\max}}} H(g_1, g_2....) P(g_1, g_2....g_{k_{\max}}) \\ &= \sum_{g_1, g_2 \cdots g_{k_{\max}}} \left(\sum_i h_i(g_i) \right) P(g_1, g_2....g_{k_{\max}}) \\ &= \sum_i \sum_{g_1, g_2 \cdots g_{k_{\max}}} h_i(g_i) P(g_1, g_2....g_{k_{\max}}) \end{aligned} \tag{3}$$

where all the sums over the variables g_i are over $g_i = 0, 1$, and the sum over i is over all edges in the metacommunity tree, i.e. all edges that *could* be in the sampled tree. In the third step we are using that the expectation value $\langle A + B \rangle$ is equal to $\langle A \rangle + \langle B \rangle$.

For each edge i we have a contribution to the expectation value

$$E(PD)_i = \sum_{g_1, g_2 \cdots g_{k_{\max}}} h_i(g_i) P(g_1, g_2 \dots g_{k_{\max}}), \tag{4}$$

which we can rewrite as:

$$E(PD)_i = \sum_{g_i} h_i(g_i) p_i(g_i) = S_i p_i(1) + 0 * p_i(0) = S_i p_i(1) \tag{5}$$

where we have introduced the marginal probability that a given edge appears in the sampled tree:

$$p_i(g_i) = \sum_{\substack{\{g_j\} \\ j \neq i}} P(g_1, g_2 \dots g_{k_{\max}}). \tag{6}$$

Finally, this gives us:

$$E(PD) = \sum_i E(PD)_i = \sum_i S_i p_i(1). \tag{7}$$

For sampling schemes such that all edges with a given number of descendent tips, k have the same marginal distribution $p_i(1) = P(k)$, we can rewrite this as

$$E(PD) = \sum_k S(k) P(k). \tag{8}$$

The function $S(k)$ is the sum over all edges with k descendent tips, which we term the Edge-length Taxa Distribution (ETD), or Edge-length Abundance Distribution (EAD) in the case of tips corresponding to individuals rather than taxa.

Sampling Schemes

A binomial sampling scheme with probability q that a given tip appears in the sampled tree leads to the marginal probability

$$P_{\text{bin}}(k) = 1 - (1-q)^k \tag{9}$$

that an edge with k descendent tips appears in the sampled tree, which leads to the following expression for expected phylogenetic diversity:

$$E(PD)_{\text{binomial}} = \sum_k S(k)(1 - (1-q)^k). \tag{10}$$

The EAD therefore performs an analogous role to that of the Species Abundance Distribution (SAD) in sampling theory based around species richness rather than phylogenetic diversity.

Other common sampling schemes include Poisson sampling (random sampling with replacement) and negative binomial:

$$P_{\text{poiss}}(k) = 1 - e^{-qk} \tag{11}$$

$$P_{\text{nb}}(k) = 1 - \left(\frac{r}{qk + r}\right)^r \tag{12}$$

The parameter r represents the departure from random sampling, with positive r indicating clustered sampling, negative r overdispersed sampling, while in the limit of $r->\infty$ the negative binomial and poisson sampling are equivalent.

Variance in Sampled Phylogenetic Diversity

$$\begin{aligned}
Var(PD) &= \sum_{g_1, g_2 \cdots g_{k_{\max}}} \left(\sum_i h(g_i)\right)^2 P(g_1, g_2 .... g_{k_{\max}}) - E(PD)^2 \\
&= \sum_{g_1, g_2 \cdots g_{k_{\max}}} \left(\sum_i h_i^2(g_i) + 2\sum_{i \neq j} h_i(g_i) h_j(g_j)\right) P(g_1, g_2 .... g_{k_{\max}}) - E(PD)^2 \\
&= \sum_i \sum_{g_i} h_i^2(g_i) p_i(g_i) + 2 \sum_{i \neq j} \sum_{g_i} \sum_{g_j} h_i(g_i) h_j(g_j) p_{ij}(g_i, g_j) \\
&- \sum_i \sum_{g_i} h_i^2(g_i) p_i(g_i)^2 - 2 \sum_{i \neq j} \sum_{g_i} \sum_{g_j} h_i(g_i) h_j(g_j) p_i(g_i) p_j(g_j) \\
&= \sum_i \sum_{g_i} h_i^2(g_i) \left(p_i(g_i) - p_i(g_i)^2\right) + \sum_{i \neq j} \sum_{g_i} \sum_{g_j} h_i(g_i) h_j(g_j) \left(p_{ij}(g_i, g_j) - p_i(g_i) p_j(g_j)\right)
\end{aligned} \tag{13}$$

where the joint probability p_{ij} is defined by:

$$p_{ij}(g_i, g_j) = \sum_{\substack{\{g_k\} \\ k \neq i,j}} P(g_1, g_2 .... g_{k_{\max}}). \tag{14}$$

Next, we note that for an edge i downstream from an edge j the hierarchical structure of a tree fixes

$$p_{ij}(1,1) = p_i(1) \tag{15}$$

and so

$$Var(PD) = \sum_i S_i^2 \left(p_i(1) - p_i(1)^2\right) + \sum_{i<j} S_i S_j \left(p_i(1) - p_i(1) p_j(1)\right) \tag{16}$$

where by $i < j$ we mean that i is downstream of j, i.e. that there is a path from j to i moving in the direction of a tip of the tree. Finally, we again assume that the sampling scheme is such

that $p_i(1)$, the marginal probability that edge i appears in the sampled tree depends only on the number of descendent tips downstream from i. Then:

$$Var(PD) = \sum_k T(k)\left(P(k) - P(k)^2\right) + 2\sum_{l<k} U(k,l)\left(P(l) - P(k)P(l)\right) \tag{17}$$

where $T(k)$ is the sum of squared edge lengths over all edges with k descendent tips, and $U(k,l)$ is the product of edge lengths with k descendent tips and downstream edge lengths with $l < k$ tips.

For realistic trees, computing $U(k,l)$ increases faster with tree-size than $T(k)$ or $S(k)$, by a factor of approximately the number of tips. To make computing the variance more tractable, we have used an approximation which serves as an upper bound on the variance:

$$Var(PD) \leq Var_{upper}(PD) = \sum_k \left[T(k) + 2V(k)\right] P(k)\left(1 - P(k)\right) \tag{18}$$

where $V(k)$ is the sum over i of the product $S_i \sum_j S_j$, where S_i is any edge with k downstream tips, and the sum over j is over all edges in the clade downstream of edge i. In other words, for a given edge i, the sum over j gives the total branch length of the corresponding downstream clade. We then use

1. $V(k) = \sum_l U(k,l)$ where $U(k,l)$ is defined as above as the product of edge lengths with k descendent tips and downstream edge lengths with $l < k$ tips.
2. For the probabilities $P(k)$ that at least one tip is sampled from a clade with k tips, and $P(l)$ that *any* subclade of this clade with $l < k$ tips has at least one tip sampled, $P(k) \geq P(l)$.

to obtain the inequality:

$$\sum_{l<k} U(k,l)P(l) \leq V(k)P(k) \tag{19}$$

and hence Eq. (18). Again, the sum over l here is for all subclades with l tips downstream of an edge with k tips. Finally we note that $V(k)$ is computationally much faster to obtain than $U(k,l)$.

Expected Phylogenetic Beta-Diversity The expected shared branch length of two randomly-drawn subtrees can be formulated in a similar way, but depends on the probability $\mathcal{P}(g_1, g_2...., g'_1, g'_2....)$of two sets of variables, $\{g_i\}$ and $\{g'_i\}$ corresponding to the two trees:

$$\begin{aligned} E(\text{Shared}) &= \sum_{\substack{g_1,g_2\cdots g_{k_{\max}} \\ g'_1,g'_2\cdots g'_{k_{\max}}}} H(g_1, g_2...., g'_1, g'_2)\mathcal{P}(g_1, g_2...., g'_1, g'_2....) \\ &= \sum_{\substack{g_1,g_2\cdots g_{k_{\max}} \\ g'_1,g'_2\cdots g'_{k_{\max}}}} \left(\sum_i h_i(g_i, g'_i)\right) P_1(g_1, g_2....)P_2(g_1, g_2....) \end{aligned} \tag{20}$$

where we have used that the trees are drawn independently and so $\mathcal{P}(g_1, g_2...., g'_1, g'_2....)$ factorizes into the probabilities defined in the previous sections, but where I have labeled these probabilities P_1 and P_2 to allow for the fact that e.g. the two trees may be of different sizes. The function $h_i(g_i, g'_i)$ is equal to S_i if both g_i and g'_i are equal to one, i.e. if both trees contain edge i, and is zero otherwise.

We can similarly express this in terms of marginal probabilities $p_{\alpha i}(g_i)$ that edge i is present in tree α,where α corresponds to tree 1 or tree 2. Then:

$$E(\text{Shared}) = \sum_i \sum_{g_i} h_i(g_i, g'_i) p_{1i}(g_i) p_{2i}(g'_i) = \sum_i S_i p_{1i}(1) p_{2i}(1). \tag{21}$$

For sampling schemes such that all edges with a given number of descendent tips, k have the same marginal distribution $p_{1i}(1) = P_1(k)$, we can rewrite this as

$$E(\text{Shared}) = \sum_k S(k) P_1(k) P_2(k). \tag{22}$$

and under binomial sampling with probabilities q_1 and q_2 of each tip being sampled we have:

$$E(\text{Shared})_{\text{binomial}} = \sum_k S(k)(1 - (1 - q_1)^k)(1 - (1 - q_2)^k). \tag{23}$$

Phylogenetic Beta Diversity and the Impact of Differing Sample Sizes This impact of sample size on studies of phylogenetic beta diversity points to a need to normalize measures of phylogenetic similarity. Taking two real communities containing n_1 and n_2 individuals, we can compute the expected shared and total branch length for two randomly sampled communities of the equivalent sizes. This gives us a way to normalize both shared branch length and total branch length separately, providing a new kind of baseline for phylogenetic diversity. Our approach is to normalize Unifrac with respect to a pair of samples drawn according to a specifed sampling scheme, and again we work with binomial sampling. Using this method, we cluster gut samples in Figure 7 (using Ward's criterion), and show that gut samples from the same subject, and in particular samples taken on consecutive days, are significantly more likely to have a normalized Unifrac score of less than 1—roughly speaking, only these consecutive samples from the same subject are more similar than random.
